# Wasp Size and Prey Load in *Cerceris fumipennis* (Hymenoptera, Crabronidae): Implications for Biosurveillance of Pest Buprestidae

**DOI:** 10.3390/insects9030086

**Published:** 2018-07-19

**Authors:** Christine A. Nalepa, Whitney G. Swink

**Affiliations:** North Carolina Department of Agriculture and Consumer Services, Plant Industry Division, 1060 Mail Service Center, Raleigh, NC 27699-1060, USA; whitney.swink@ncagr.gov

**Keywords:** nest provisioning, prey, *Agrilus*, emerald ash borer, flight load, insect survey, invasive pests

## Abstract

The relationship between predator and prey size was studied in the buprestid hunting wasp *Cerceris fumipennis* Say in eight widely distributed nesting aggregations in North Carolina, USA. Initial work indicated a significant linear relationship between wasp head width and wasp wet weight; thus, head width was used to estimate wasp body mass in subsequent studies. Prey loads of hunting females was studied by measuring the head width of the wasp, then identifying and weighing the prey item brought back to the nest. There was significant variation in wasp size among nesting aggregations; the average estimated wasp body mass in one site was double that in another. Prey weight varied with wasp weight, but larger wasps had a slight tendency to carry proportionally larger prey. Beetles captured by large wasps (≥120 mg) were significantly more variable in weight than those taken by small wasps (<80 mg). All but the smallest wasps could carry more than their own body weight. Prey loads ranged from 4.8–150.2% of wasp weight. Evidence suggests that small wasps bring back more of the economically important buprestid genus *Agrilus* and thus would be most efficient in biosurveillance for pest buprestids.

## 1. Introduction

*Cerceris fumipennis* Say is a solitary, ground-nesting wasp that typically nests in aggregations. Females provision their nest with beetles in the family Buprestidae, which they capture in nearby vegetation, paralyze, and transport back to their nest as food for their offspring. Because these wasps collect such a wide range of buprestid species as prey, they are being used in North America as a biosurveillance tool that complements trapping surveys for detecting nonindigenous buprestid beetles of current or potential concern. Most of these invasives are in the genus *Agrilus* Curtis, and include the emerald ash borer (*Agrilus planipennis* Fairmaire), European oak borer (*A. sulcicollis* Lacordaire), goldspotted oak borer (*A. auroguttatus* Schaeffer), and oak splendor beetle (*A. biguttatus* (Fab.)) [1,2,3,4]. The wasp is known to be particularly effective at detecting these pests at low densities [5].

The current study on *C. fumipennis* body size is rooted in the well-established principle that the major factor limiting capture and transport of prey in hunting wasps is the size of the female [6,7]. Like many other sphecoid wasps, *C. fumipennis* is a single-prey loader, i.e., they carry one item per hunting trip back to the nest. This species is known to utilize a wide size range of beetles as prey compared to other species in the genus. For example, *Cerceris halone* Banks hunts prey weevils that range between 5.5–7.5 mm in length (see [8]: Figure 5). *Cerceris fumipennis,* on the other hand, transports back to her nest buprestid beetles ranging from 4.9 to 21.3 mm in length [9]. The size relationship between *C. fumipennis* and her prey has been addressed in the past; however, the measure of prey size in these studies typically utilized body length, which is not always an appropriate measure of buprestid body mass because body shape varies substantially among genera [9]. In North Carolina, more than 72 buprestid species have been identified as *C. fumipennis* prey since 2009 ([2]; W.G. Swink, unpublished data), ranging from the robust, oblong bodies of *Buprestis* L., to the squat, shield-shaped bodies of *Brachys* Dejean and the elongate parallel-sided, generally small bodies of *Agrilus* (see [10]). Rather than using body length of prey, then, we explored the relationship between wasp weight and prey weight, and concentrated our analysis on beetles of the genus *Agrilus*.

It is known that head width is correlated with body weight in studied sphecoid wasps (e.g., [7,11]), and head width has been used to determine body size in *C. fumipennis* [12]; our initial study was designed to confirm that correlation. A strong linear relationship between the two would support the use of head width as an appropriate estimate of wasp body mass in field studies. The second aim was to explore the relationship between wasp body size and the size and identity of buprestid beetles transported back to the nest in eight widely distributed nesting aggregations.

## 2. Materials and Methods

An examination of the relationship between head capsule width and body mass of *C. fumipennis* was conducted in 2013. The study site was a nesting aggregation on a softball diamond at a private school in Goldsboro, Wayne Co., North Carolina (35.398° N, 78.013° W). On five days during the nest-founding phase, between 2 June and 15 June, attempts were made to capture all wasps flying on the field, regardless of whether they were recurrently associated with a nest. Netted wasps were confirmed as females by their facial markings, and then weighed after tightly confining them in a small, tared, zip-top plastic bag. Their head capsule width was measured, and, to avoid duplicate measurements, each was given a distinctive mark by attaching a small numbered plastic disc to the thorax (Queen Marking Kit; The Bee Works, Orillia, ON, Canada) with a dot of Loctite^®^ Super Glue (Henkel Corporation, Westlake, OH, USA). The wasp was then released near her point of capture (n = 64). Weights were recorded in the field using an Ohaus Scout Pro balance (±0.001 g) (Ohaus Corporation, Pine Brook, NJ, USA). Each female was weighed three times and the average fresh weight recorded. Wasp head width was taken using a Mitutoyo Absolute Digimatic Caliper (0.01 mm) (Mitutoyo Corporation, Kanagawa, Japan); as with the weights, three measurements were taken and the average recorded.

In 2014 we worked in eight nesting aggregations of *C. fumipennis* (Table 1) across North Carolina ranging from the coastal plain to the mountains. At each site, female wasps returning from a successful hunting trip were captured using methods previously described [1,2,5,13], their head capsule width measured as before, and then released. The prey each was carrying was collected and transported on ice to the laboratory, where the beetles were weighed using an Ohaus^®^ Explorer balance (0.0001 g) (Ohaus Corporation, Florham Park, NJ, USA). Beetle fresh weight was used in all analyses of this study, which commenced on 28 May, and terminated 8 July, 2014. *Cerceris fumipennis* is thought to be univoltine in North Carolina [14].

Buprestidae were identified by Whitney G. Swink and Joshua Basham. The 2013 data were analyzed using linear regression. Prey load was defined as (prey weight/predicted wasp weight) × 100. The 2014 data were normalized using Johnson Sb transformation. Bartlett’s test of wasp size indicated variances were not significantly different among sites (*p* > 0.05); data were analyzed using analysis of variance in SYSTAT v.13.1 (Systat Software, Inc., Chicago, IL, USA), and Tukey’s test used for post-hoc comparisons among sites.

## 3. Results

**Figure 1:**
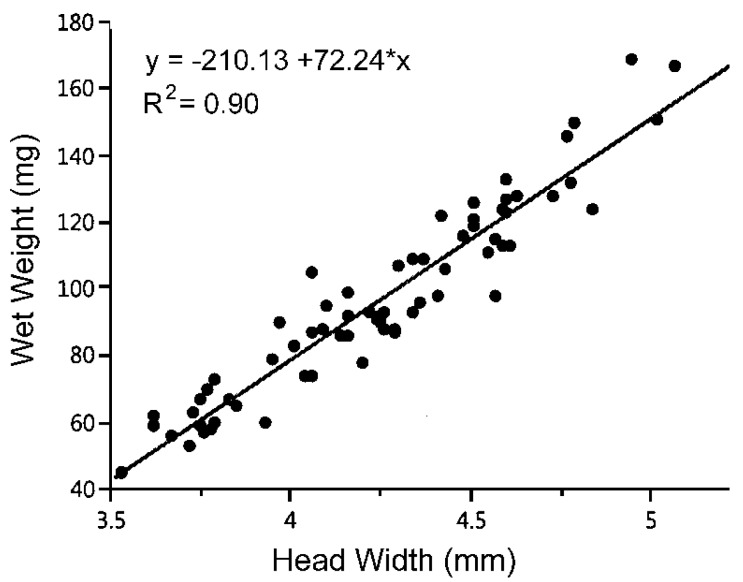
A linear relationship between head width and body mass (wet weight) in *Cerceris fumipennis* (*P* < 0.001) at one North Carolina site.

In the 2013 preliminary study, the head capsule width of female *C. fumipennis* at the site was significantly related to wet weight (*R*^2^ = 0.90; *p* < 0.0001) (Figure 1). The measure of head capsule width was therefore an appropriate measure of body size in the wasp, and we calculated the predicted wet weight from wasp head capsule measurements using the regression equation in Figure 1 for subsequent analyses. In 2014, data on a total of 258 wasps and their prey were collected from the eight investigated sites (Table 2), with sample sizes ranging from 10–51 per site. Predicted wasp weight averaged 103.1 mg, but ranged from 29.7 to 159.0 mg and displayed significant differences among sites (*F*_7,249_ = 26.139; *p* < 0.0001). The average predicted weight of wasps in Site 1 was more than double that of those at Site 8; wasp weights at the remaining six sites were not significantly different from each other (Table 2). Head capsule widths of the wasps in Site 1 (range 4.22–5.09 mm) did not overlap with head capsule widths of the wasps in Site 8 (range 3.32–4.17 mm).

**Figure 2:**
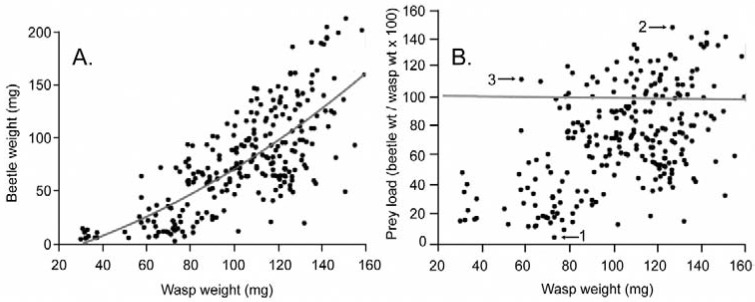
Relationship between predicted wasp weight and prey beetle weight in hunting *Cerceris fumipennis*. (**A**) Prey beetle weight as a function of wasp weight. The relationship is best described with the equation: Beetle wt. (mg) = –59.19378 + 1.3317934 * Wasp Wt. (mg) + 0.0037293 * (Wasp Wt. (mg) − 103.066)^2^; R = 0.55, *p* < 0.0001. (**B**) Percent body weight (prey load) carried by *C. fumipennis* females ((beetle weight/wasp weight) × 100)). Points above the line indicate females carrying more than their own body weight. Data point 1: smallest prey load (4.8%, a wasp weighing 72.3 mg carrying a 3.5 mg *Agrilus subrobustus*); data point 2: largest prey load (150.2%, a wasp weighing 125.8 mg carrying a 188.9 mg *Buprestis rufipes)*; data point 3: smallest wasp carrying more than her own body weight (113.6% prey load, a wasp weighing 57.2 mg carrying a 65.0 mg *Buprestis maculipennis*).

Overall, collected beetle prey were in the genera *Actenodes* Dejean, *Agrilus*, *Brachys*, *Buprestis*, *Chrysobothris* Eschscholtz, *Dicerca* Eschscholtz, *Eupristocerus* Deyrolle, *Phaenops* Dejean, and *Spectralia* Casey. Prey weight generally varied with wasp weight (range of beetle weight: 3.5 to 216.4 mg; Table 2). A quadratic equation yields a marginally better fit of the data than a linear regression (*R*^2^ = 0.552 and 0.547, respectively), indicating that there is a slight tendency for large wasps to carry proportionally larger prey (Figure 2a). All but the smallest wasp females could carry more than their own body weight (Figure 2b); prey load averaged 73.8% and ranged from 4.8% (*Agrilus subrobustus* Saunders) to 150.2% (*Buprestis rufipes* Olivier) of wasp body weight. Note that it wasn’t the largest wasp that carried the highest prey load (Figure 2b, data point 2). *Cerceris fumipennis* females occasionally collect a mating pair of beetles [5,15]; just one mating pair of beetles was collected during this study, a male and female *Buprestis maculipennis* Gory that together were 111% of the wet weight of the wasp carrying them.

Of special interest are wasps that collected beetles in the economically important genus *Agrilus.* At least one beetle in the genus *Agrilus* was brought back to the nest at seven of the eight sites we monitored. The 27 *Agrilus* collected averaged 13.5 ± 7.7 mg, and ranged from 3.5–33.0 mg in weight. The wasps that collected these *Agrilus* averaged 62.6 ± 22.4 mg (range: 29.7–131.6 mg). It should be noted that the largest wasp (131.6 mg) to bring back *Agrilus* was an anomaly; the remaining 26 wasps ranged from 29.7 to 86.8 mg. No *Agrilus* were collected at Site 1, where wasps were significantly larger than all but two of the remaining sites and 92.3% of beetles collected were in the genus *Buprestis.* The most *Agrilus* (48%) were collected at Site 8, where wasps were significantly smaller than in the remaining seven sites.

To further explore the relationship between *Agrilus* retrieval and wasp size, we compared the prey of large (≥120 mg) vs. small (≤80 mg) wasps over the eight sites. Large wasps were found in all sites but Site 8; small wasps were absent in Sites 1 and 5. Although more total beetles were captured by those classified as large wasps during this study, small wasps captured more beetle species than the large (n = 12 and 9 species, respectively; Table 3). Beetles taken by large and small wasps weighed an average of 116 ± 46.1 and 29.7 ± 24.8 mg, respectively, but beetles captured by large wasps were significantly more variable in weight (Brown–Forsythe, *F*_1,138_ = 24.3, *P* < 0.001). The range of prey size of large wasps was more than double that of the small (a weight spread of 194 and 93 mg, respectively; Table 3). The range of prey loads in the two wasp size categories, however, was more similar (prey load spread of 133 and 118%, respectively). Just one *Agrilus* was captured by large *C. fumipennis*, but 26 *Agrilus* in seven species were captured by the small wasps (Table 3). *Buprestis maculipennis* was the sole beetle species taken as prey by both wasp size categories. *Buprestis maculipennis* taken by small wasps weighed significantly less than those taken by large wasps (mean of 87.9 ± 28.7 and 72.4 ± 11.0 mg, respectively; *F*_1,44_ = 8.0, *p* < 0.01, Welch’s *t*-test).

## 4. Discussion

As in other studied *Cerceris* species (e.g., [6,8,16]), the present work demonstrates that, although large *C. fumipennis* females typically provision their nests with buprestids larger than those taken by small females, they will accept prey beetles that vary over a wide size range. Small wasps, on the other hand, are mechanically constrained from hunting large prey [17,18]. Overall, the average *C. fumipennis* prey load was 74% of wasp body mass. The maximum was 150% of body weight for large wasps, but a beetle just 17% percent of wasp body weight was nonetheless acceptable. The range of prey loads found in small wasps was 5–122%. There may exist a distinct switch-point in wasp size, above which wasps begin to hunt larger prey [19]. If so, it may occur at about 60 mg in *C. fumipennis*, as that is when they begin carrying beetles above their own body weight (Figure 2b, data point 3).

Prey length that has been reported for *C. fumipennis* varies most noticeably in the upper range limits. Evans and Rubink [20] reported that prey ranged from 5.5 to 10.5 mm in Texas; similarly small prey (4.2–12.0 mm) were taken by *C. fumipennis* in British Columbia [3]. Lengthier beetles (4.1–18.9 mm) were included in the prey menu of *C. fumipennis* in New York [21] and North Carolina (4.9 to 21.3 mm), where just 21.7% of the beetles collected in the state fell into the range reported by Evans and Rubink [20] (see [9]: Figure 1). This reported variation in prey length is likely related to significant differences in wasp size that occur at the population level (Table 2). For example, 91.4% of the prey beetles collected at our Site 8 measured less than the 10.5 mm maximum reported in the Evans and Rubink [20] study, and the wasps at Site 8 were significantly smaller than in the other seven sampled populations. 

It is well established that wasp size determines the upper size limit of prey that can be carried in flight [3,6,22,23]. A more complex issue centers on the determinants of wasp size, which this study demonstrates can vary dramatically among nesting aggregations. In their studies of the cicada killer wasp *Sphecius speciosus* Drury, Hastings et al. [24] initially suggested that size differences in their studied wasps reflected the relative abundance of differently-sized prey at the locations they investigated. Later, however, they indicated that if sampled prey are small, it is because the wasps in a given location are small, not because small prey predominate there [19].

Regardless of the size of prey beetles available near a nesting area, maternal decisions play a major role in determining the size of the next wasp generation. The amount of food allocated to a given wasp larva is fixed when the mother closes the brood cell, and sets an upper limit to the final body size of offspring. In *C. fumipennis*, the number of prey a female provides to a cell is inversely related to the size of available prey [25]. In a population examined in Texas [15], the number of beetles per cell ranged from three large *Descarpentriesina cyanipes* (Say) to 51 small *Agrilus* species, indicating flexibility in provisioning behavior and a degree of control over offspring size. What, though, is the basis for maternal decision-making? Because body size and reproductive success are usually positively correlated [26], the optimal size daughter should be the largest size possible [27]. In this case, however, reproductive success may be maximized by making daughters just large enough to handle the prevailing size of prey available near the nesting site. Body size, egg size, and provisioning behavior also may have some direct heritability in digging wasps [11,27]. Willmer [28] additionally suggested that small tunnels made by the mother may trap large daughters when they try to emerge, and that accumulating the large number of prey required to produce a large daughter may increase levels of parasitism. The former suggestion seems unlikely, as at least some emerging brood dig out vertically from their brood cells instead of utilizing the natal nesting tunnel (Figure S1).

Although each of these factors may play some role in determining the size of females that emerge at the beginning of the nesting season, it is rarely considered that postemergence behavior may play a major role in regulating the size of *C. fumipennis* females that remain in a nesting aggregation. The average size of females holding a nest increases during the nest-founding stage, because large females competitively displace smaller females from their nests [12]. Thus, as small wasps emerge, they may be filtered from the aggregation depending on their ability to hold a nest. Small females also may choose to nest elsewhere if most prey locally available are above the size threshold they can handle. Conversely, a large wasp may emigrate if small buprestids dominate in trees near the nesting area. Testing these ideas would require establishing the relative availability of different size prey in the vicinity of a given nesting aggregation. That, however, would be difficult, as the majority of these prey beetles frequent tree canopies, out of reach and out of sight. Nonetheless, evidence for postemergence mobility of the wasps and their aptitude for becoming established in new areas was provided by Johnson et al. [4], who found numerous nesting aggregations in forest clearings that had been heavily vegetated and devoid of nests the previous year, indicating that these wasps emerged elsewhere. 

## 5. Conclusions

Whatever the core reason for the significant difference in wasp size we found among sites, it seems clear that, from a practical viewpoint, an aggregation made up of small wasp females would be maximally effective at collecting the economically important *Agrilus* species that are the intended targets of biosurveillance. Although large wasps exploited a broader range of prey size, small *C. fumipennis* females brought back a broader taxonomic spectrum of prey (Table 3), primarily due to the number of *Agrilus* species captured. Utilizing populations of small wasps for biosurveillance, then, would maximize detection of potential pests. Support for this idea comes from Swink et al. [29], who detailed the first collection of the East Asian buprestid *Agrilus subrobustus* Saunders from Site 8 of this study, where females were significantly smaller than the other seven examined sites. Nonetheless, we acknowledge that, given the difficulty of finding nesting aggregations of *C. fumipennis* wasps of any size in North Carolina [30], limiting biosurveillance efforts to sites with small wasps would be strongly restrictive.

## Figures and Tables

**Table 1:** North Carolina counties, sites, and dates visited during the 2014 study of *Cerceris fumipennis* foraging behavior.

Dates	County	Site Number and Name	Site Coordinates
28 May–6 June	Wayne	1. Wayne Comm College	35.402° N, 77.942° W
31 May–11 June	Wayne	2. Faith Christian Academy	35.398° N, 78.013° W
9 June–14 June	Franklin	3. Franklinton Park	36.108° N, 78.437° W
11 June–15 June	Wake	4. Lake Lynn	35.889° N, 78.698° W
16 June–25 June	Surry	5. Meadowview Middle School	36.481° N, 80.652° W
19 June–23 June	Alamance	6. McCray Park	36.171° N, 79.386° W
27 June–8 July	Franklin	7. Luddy Park	36.023° N, 78.483° W
24 June–2 July	Buncombe	8. Vance Elementary	35.577° N, 82.600° W

**Table 2:** Mean predicted wasp weight, prey weight, and prey load (% body weight: (beetle weight/wasp weight) × 100) at eight study sites in North Carolina. Site numbers are ordered by wasp weight; mean wasp weights followed by different letters are significantly different at *p* < 0.05.

Site	Sample Size (n)	Mean ± SD Predicted Wasp Wet Weight (mg)	Mean ± SD Prey Beetle Wet Weight (mg)	Minimum/Maximum Beetle Wet Weight (mg)	Mean ± SD % Body Weight Carried by Wasp	Range % Body Weight Carried by Wasp
1	26	126.21 ± 14.86 a	109.11 ± 49.29	32.8–205.6	85.07 ± 33.85	34.6–150.2%
6	10	118.67 ± 32.45 a,b	91.89 ± 5 8.84	7.8–192.3	71.18 ± 40.22	10.2–146.6%
7	51	113.31 ± 23.51 a,b	95.64 ± 51.15	12.5–216.4	81.22 ± 35.33	
2	45	107.99 ± 21.34 b	84.68 ± 38.00	7.8–166.9	76.65 ± 28.36	
5	11	103.86 ± 14.19 b	92.82 ± 38.25	17.2–149.8	87.70 ± 32.83	
4	47	103.00 ± 20.69 b	85.31 ± 40.23	6.0–163.9	79.49 ± 32.53	
3	33	101.28 ± 21.78 b	78.15 ± 37.39	10.4–202.3	76.80 ± 29.95	
8	35	61.67 ± 18.80 c	23.25 ± 16.00	3.5–70.1	36.59 ± 20.14	4.8–94.1%
**Overall**	258	103.07 ± 27.51	80.88 ± 47.59	3.5–216.4	73.76 ± 34.27	4.8–150.2%

**Table 3:** Comparison of the identity, number, and weight of prey Buprestidae collected by large (≥120 mg) vs. small (≤80 mg) *Cerceris fumipennis* at eight study sites (pooled) in North Carolina.

Large Wasp (≥120 mg) Prey Species	n	Small Wasp (≤80 mg) Prey Species	n
*Agrilus ferrisi* Dury	1	*Agrilus acornis* (Say)	2
*Buprestis consularis* Gory	7	*Agrilus arcuatus* (Say)	5
*Buprestis lineata* F.	16	*Agrilus bilineatus* (Weber)	12
*Buprestis maculipennis* Gory	44	*Agrilus cliftoni* Knull	1
*Buprestis rufipes* Olivier	9	*Agrilus difficilis* Gory	1
*Buprestis striata* F.	1	*Agrilus ruficollis* (F.)	4
*Chrysobothris dentipes* (Germar)	1	*Agrilus subrobustus* Saunders	1
*Chrysobothris shawnee* Wellso and Manley	1	*Brachys ovatus* (Weber)	3
*Dicerca lurida* (F.)	3	*Buprestis maculipennis* Gory	11
		*Chrysobothris sexsignata* Say	12
		*Eupristocerus cogitans* (Weber)	2
		*Phaenops aeneola* (Melsheimer)	3
Total beetles	83		57
Mean ± SD weight of beetles (mg)	116.0 ± 46.1		29.7 ± 24.8
Range weight of beetles (mg)	22.1–216.4		3.5–95.6
Range prey load (%)	16.8–150.2		4.8–122.4

## Supplementary Materials

The following are available online at http://www.mdpi.com/2075-4450/9/3/86/s1, Figure S1: Emergence of *Cerceris fumipennis* at the beginning of the nesting season. One female has emerged from and taken over the main tunnel of the nest established by her mother (left); others, possibly males, have emerged vertically from their brood cells (n = 4, right).
