# Supplementary material for: Wasp Size and Prey Load in Cerceris fumipennis (Hymenoptera, Crabronidae): Implications for Biosurveillance of Pest Buprestidae
Source: Insects. 2018 Jul 19;9(3):86. doi: 10.3390/insects9030086 (PMC6164872; doi:10.3390/insects9030086)
Supplement: Supplementary file 1 [file insects-09-00086-s001.pdf]

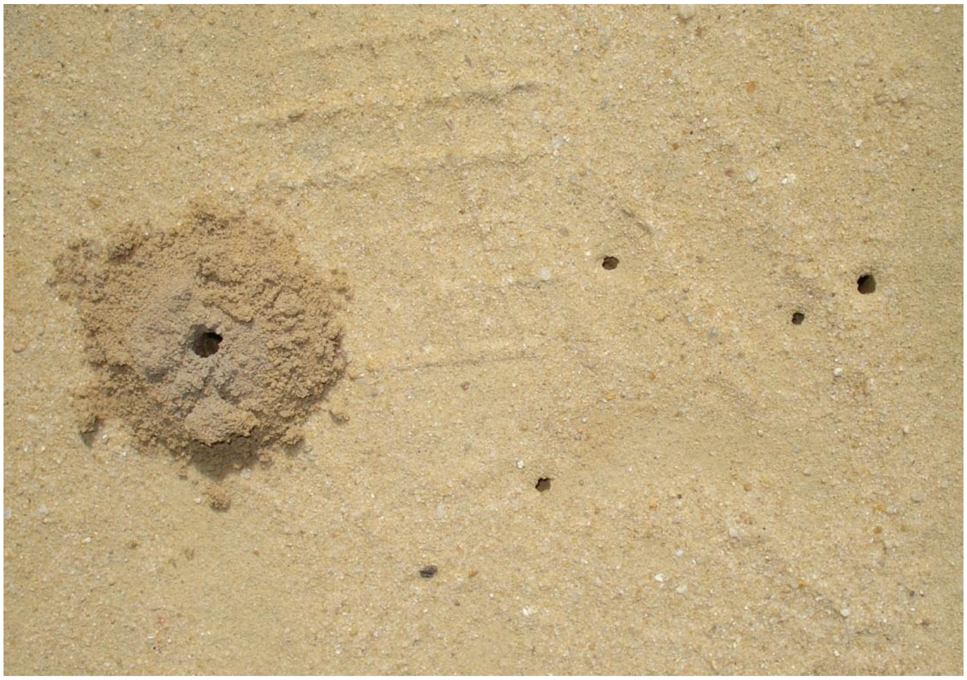

Figure S1. Emergence of *Cerceris fumipennis* at the beginning of the nesting season. One female has emerged from and taken over the main tunnel of the nest established by her mother (left); others, possibly males, have emerged vertically from their brood cells (n = 4, right).
